# The Signal Sequence Influences Post-Translational ER Translocation at Distinct Stages

**DOI:** 10.1371/journal.pone.0075394

**Published:** 2013-10-09

**Authors:** Nicholas Johnson, Sarah Haßdenteufel, Melanie Theis, Adrienne W. Paton, James C. Paton, Richard Zimmermann, Stephen High

**Affiliations:** 1 Faculty of Life Sciences, University of Manchester, Manchester, United Kingdom; 2 Medical Biochemistry and Molecular Biology, Saarland University, Homburg, Germany; 3 Research Centre for Infectious Disease, University of Adelaide, Adelaide, Australia; 4 Institute of Organic Chemistry and Biochemistry, Academy of Sciences of the Czech Republic, Prague, Czech Republic; University of Toronto, Canada

## Abstract

The metazoan Sec61 translocon transports polypeptides into and across the membrane of the endoplasmic reticulum via two major routes, a well-established co-translational pathway and a post-translational alternative. We have used two model substrates to explore the elements of a secretory protein precursor that preferentially direct it towards a co- or post-translational pathway for ER translocation. Having first determined the capacity of precursors to enter ER derived microsomes post-translationally, we then exploited semi-permeabilized mammalian cells specifically depleted of key membrane components using siRNA to address their contribution to the membrane translocation process. These studies suggest precursor chain length is a key factor in the post-translational translocation at the mammalian ER, and identify Sec62 and Sec63 as important components acting on this route. This role for Sec62 and Sec63 is independent of the signal sequence that delivers the precursor to the ER. However, the signal sequence can influence the subsequent membrane translocation process, conferring sensitivity to a small molecule inhibitor and dictating reliance on the molecular chaperone BiP. Our data support a model where secretory protein precursors that fail to engage the signal recognition particle, for example because they are short, are delivered to the ER membrane via a distinct route that is dependent upon both Sec62 and Sec63. Although this requirement for Sec62 and Sec63 is unaffected by the specific signal sequence that delivers a precursor to the ER, this region can influence subsequent events, including both Sec61 mediated transport and the importance of BiP for membrane translocation. Taken together, our data suggest that an ER signal sequence can regulate specific aspects of Sec61 mediated membrane translocation at a stage following Sec62/Sec63 dependent ER delivery.

## Introduction

In eukaryotes, secretory proteins synthesized in the cytosol must first translocate across the membrane of the endoplasmic reticulum (ER) to enter the secretory pathway. The precursors for these proteins are characterized by a sequence of hydrophobic amino acids at their N-terminus that identifies them for targeting to the ER [1], [2]. This signal sequence is recognized by targeting factors in the cytosol, in many cases via the actions of the signal recognition particle (SRP), which targets the precursor to the ER membrane in the form of a ribosome/nascent chain complex [2]. This delivery step relies on interactions with the SRP receptor and, after the release of the signal sequence from SRP, the nascent chain is extruded into the ER lumen through the Sec61 translocon coupling translocation across the ER membrane to ongoing translation at the ribosome [3]. After this initial engagement of the nascent chain with the ER translocon, current models suggest that the hydrophobic signal sequence exits the Sec61 complex via a lateral gate. This provides access to the signal peptidase complex that removes the ER targeting signal, often as protein synthesis continues, enabling the mature domain to achieve a native conformation and progress through the secretory pathway [4].

In contrast to this co-translational pathway for ER translocation, some secretory proteins can be targeted to the ER after their synthesis is complete, resulting in their translocation being ribosome independent and post-translational [5]. The process of post-translational protein translocation across the ER membrane is well documented in *Saccharomyces cerevisiae*, where several model substrates that exploit this process, including prepro-α-factor (PpαF), have been studied extensively [6]. Importantly, secretory protein precursors that employ this post-translational route possess well-defined hydrophobic N-terminal signal sequences that are responsible for their delivery to the ER. However, in yeast it appears that the signal sequences carried by precursors that favor a post-translational pathway for translocation are less hydrophobic than those of SRP dependent precursors, providing a rationale for their failure to engage SRP [7], [8]. In the case of PpαF, the completed polypeptides are prevented from aggregating in the cytosol by Hsp40/Hsp70 chaperones that maintain them in a loosely folded or “translocation competent” state [9]. Once at the ER membrane the signal sequence binds to the yeast ER translocation complex, consisting of the Sec61 translocon and the associated Sec62/63/71/72 complex [10], [11]. The efficient post-translational translocation of the mature domain of the precursor across the membrane is also facilitated by the ER luminal Hsp70 chaperone, Kar2 [12]. Strikingly, a recent study has suggested that >40% of *S. cerevisiae* precursors reach the ER independently of SRP, including GPI anchored proteins, where efficient delivery relies on both distinct Hsp40 family members and the ATP dependent targeting factor, Get3 [8].

It is becoming increasingly apparent that post-translational translocation across the ER membrane is not limited to lower eukaryotes. Hence, metazoans secrete many small proteins that function as hormones, chemokines and antimicrobial peptides, and their precursors are often simply too short to efficiently engage SRP and exploit a co-translational biosynthetic pathway [13]. Intriguingly, the signal sequences of such proteins can be artificially induced to bind SRP by extending their C-termini, increasing the opportunity for SRP to bind co-translationally to the emerging signal sequence [14]. In practice, short secretory proteins like preprocecropin A (PpCecA) can utilise multiple pathways for their post-translational delivery to the ER [15], [16], apparently mirroring the complexity of SRP independent targeting in yeast [8]. One of the pathways implicated in the biogenesis of short secretory proteins involves TRC40 [16], the metazoan homologue of Get3 that mediates the post-translational insertion of tail-anchored (TA) membrane proteins at the ER [17]. Likewise, calmodulin has also been suggested to deliver short secretory proteins, including PpCecA, to the ER, in this case in a calcium dependent fashion [15]. Notably, both calmodulin and TRC40 bind directly to the signal sequence of short secretory proteins, effectively shielding this hydrophobic region in the cytosol during precursor delivery to the ER [15], [16], a function that seems to be an important element of such post-translational ER delivery factors [8]. Upon arrival at the mammalian ER, the completed precursor proteins are translocated across its membrane via the Sec61 complex [16], [18]. However, two other key components, Sec62 and Sec63, are also required for this post-translational process to be effective [18], [19].

In this study, we have investigated the role of the N-terminal signal sequence during post-translational translocation across the ER membrane focusing on two specific issues: i) is the signal sequence the sole parameter that enables post-translational translocation; ii) can the signal sequence influence the post-translational pathway that a substrate follows at the translocon? We find that a signal sequence alone is not sufficient to confer the capacity for efficient post-translational translocation of a polypeptide chain, and show that the mature domain has a strong influence. The signal sequence does however appear to dictate the precise pathway by which a polypeptide engages the ER translocon. We speculate that whilst precursors may be transferred to Sec61 by different mechanisms and accessory components, the requirements for the subsequent translocation event is dictated by the properties of the signal sequence irrespective of the upstream delivery pathway to the ER membrane.

## Materials and Methods

### Materials

Nuclease-treated rabbit reticulocyte lysate for in vitro translation was obtained from Promega. The ER translocation inhibitor, CPD A, was kindly provided by Novartis and was prepared by dissolving in DMSO and storing at −80°C. ER microsomes were prepared from dog pancreas as previously described [20].

### cDNAs and Transcription

Opsin tagged PpCecA constructs, PpCecAOPG1 and PpCecAOPG2, are based on the protein from the Cecropia moth (UniProt ID: P01507) and have been described previously [16]. The signal sequence (amino acids 1–20) of the Saccharomyces cerevisiae protein PpαF (UniProt ID: P01149) was added to the mature domain of cecropin and the signal sequence of PpCecA (amino acids 1–22) was added to the mature region of α-factor by PCR to generate PαFpCecAOPG1 and PCecApαF respectively. These coding regions were cloned into the pTNT vector (Promega) for in vitro translation. Transcripts were synthesised from PCR derived templates using T7 RNA polymerase with the exception of PpαF, which was synthesised using SP6 polymerase from the SP65 vector (Promega).

### *In vitro* Translation and Membrane Translocation

**Figure 1:**
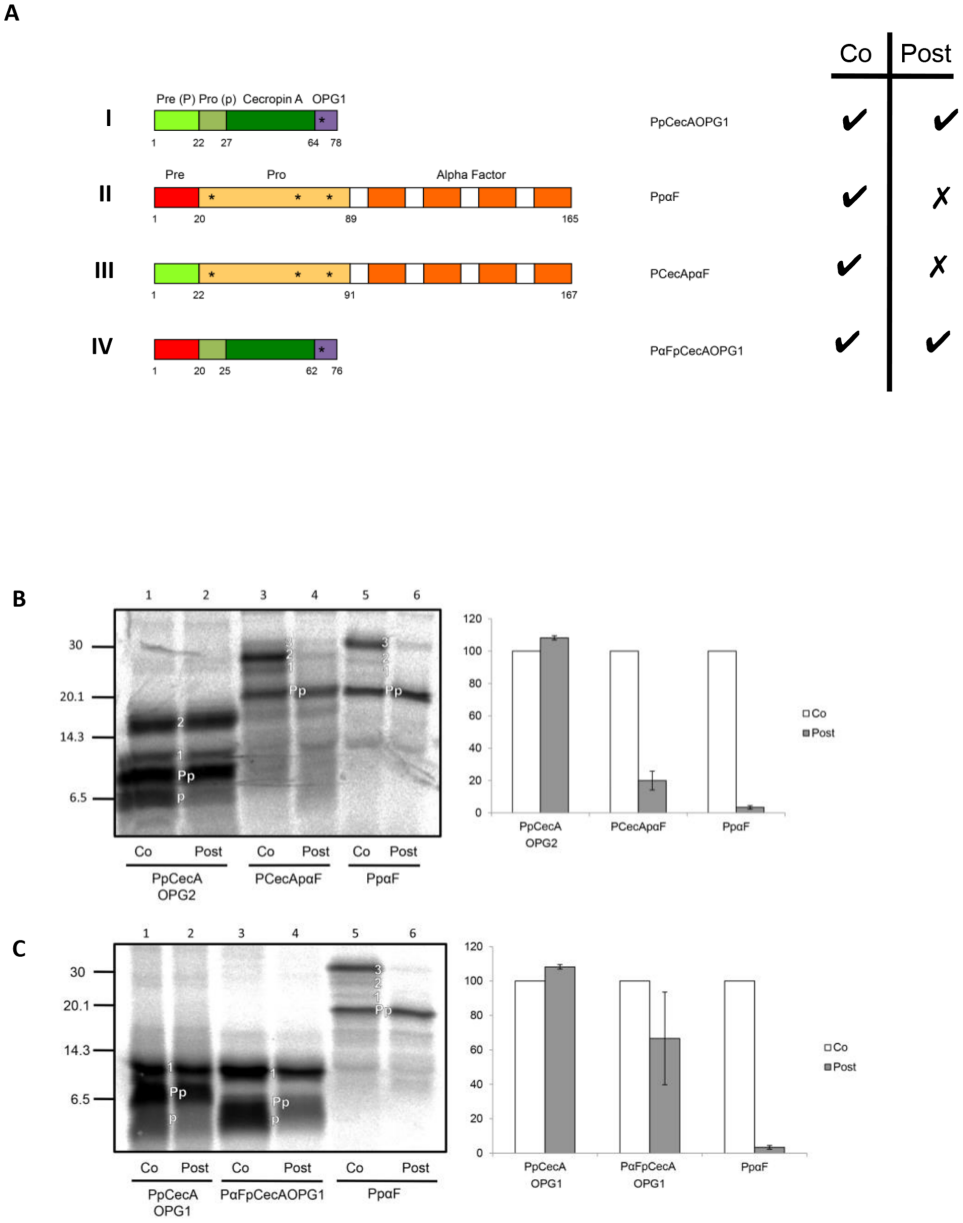
Signal sequence chimeras of PpCecA and PpαF. **A**. Schematic representation of proteins derived from PpCecA and PpαF. Signal sequences (denoted Pre or P) are in light green and red respectively, whilst the purple region denotes an opsin epitope tag bearing one N-glycosylation site (OPG1). The OPG2 tag contains an additional four residues that includes a second N-glycosylation site, and both PpCecAOPG1 and PpCecAOPG2 are as previously described [16]. Numbers indicate first and last amino acids of each region, and asterisks indicate the approximate location of sites for N-linked glycosylation. The ability for each protein to exploit co- and post-translational translocation pathways, as deduced from the data shown in panels B and C, is indicated by a tick. **B**. The PpCecA signal sequence is not sufficient to enable efficient post-translational translocation of PpαF. The precursors indicated were synthesized in vitro using rabbit reticulocyte lysate and [^35^S] methionine in either the presence of canine pancreatic microsomes (co) or prior to their addition (post). The membrane fraction was isolated by centrifugation and associated proteins analyzed by SDS-PAGE and phosphorimaging. The untranslocated (Pp), singly, doubly and triply N-glycosylated (1, 2, 3) and signal cleaved (p) forms of the precursors are indicated (see also Figs. S1 and S2). The amount of post-translationally translocated material as a proportion of the total membrane associated material was quantified and is expressed relative to the proportion of material translocated under co-translational conditions (set to 100%). Results are given as means (±s.e) for n = 3. **C**. PpCecA can efficiently translocate post-translationally with the PpαF signal sequence. The precursors indicated were synthesized and the resulting data analyzed as described for **B**, with results given as means (±s.e) for n = 3.

Proteins were synthesised from RNA transcripts in 20 µl reactions using nuclease-treated rabbit reticulocyte lysate. Translations were performed in the presence of [^35^S]-methionine (0.615 MBq per 20 µl reaction, specific activity 43.48 TBq/mmol) for at least 15 minutes at 30°C. Unless otherwise stated, ER translocation assays were carried out “co-translationally”, as the model proteins were translated in the presence of 10% v/v of an ER microsome suspension (40 A_280_/ml), or 24% v/v (for PpCecAOPG1 and PαFpCecAOPG1) and 32% v/v (for PpαF and PCecApαF) of a semi-intact cell suspension (40,000 cells/µl). Membrane translocation experiments utilizing semi-intact cells depleted of membrane components have been previously described [18]. For post-translational assays (e.g. Figs. 1B and 1C), protein synthesis was performed in the absence of any added membrane, puromycin added to 1 mM and the reaction incubated for a further 5 minutes at 30°C to release of polypeptide chains from the ribosome and then ER derived microsomes (10% v/v) added and samples incubated for 20 minutes at 30°C to enable translocation.

Following membrane tranlocation, ER derived microsomes were recovered by centrifugation through 120 µl HSC (750 mM sucrose, 500 mM KOAc, 5 mM Mg(OAc)_2_, 50 mM Hepes-KOH pH 7.9) at 100,000×*g* for 10 minutes, whilst semi-intact cells were re-isolated by centrifugation at 40,000×*g* for 20 minutes. The membrane pellet was resuspended in 20 µl LSC (100 mM sucrose, 100 mM KOAc, 5 mM Mg(OAc)_2_, 50 mM Hepes-KOH pH 7.9, 1 mM DTT) and treated with 250 µg/ml RNase A at 37°C for 10 minutes to remove any residual peptidyl-tRNA species. The resulting samples were analysed by SDS-PAGE and phosphorimaging. Comparisons of the relative efficiency of protein translocation or membrane insertion are based on the quantification of N-glycosylated species or signal cleaved species. Unless otherwise stated, the major glycoform (or signal cleaved form) of a model protein was quantified for each of the experimental conditions indicated, and the resulting values expressed as a percentage of the relevant matched control (set to 100%). Quantification was performed as previously described [16] using either Aida or Image Quant 5.0 software.

### CPD A and SubAB Treatment

CPD A was added to the translation reaction to a concentration of 10 µM. In co-translational assays, the compound was present during synthesis whereas for post-translational assays, it was added after synthesis but prior to addition of membranes. Recombinant SubAB (cytotoxin AB_5_ subtilase) and SubA_A272_B were purified as His6-tagged fusion proteins by Ni-NTA chromatography as previously described [21]. To carry out assays under conditions where BiP activity was inhibited [22], cells were pre-incubated with SubAB, or the inactive mutant SubA_A272_B, at 1 µg/µl and 37°C for 2 hours prior to their semi-permeabilization for use in ER translocation assays.

## Results

### Signal Sequence Exchange does not Affect Capacity for Post-translational Translocation

In order to investigate the role of the signal sequence in post-translational translocation, we constructed a chimera in which the signal sequence of PpαF (Fig. 1A, II) was replaced by that of PpCecA (Fig. 1A, I), and vice versa, generating the chimeric proteins; PCecApαF and PαFpCecA (Fig. 1A, III and IV). Interestingly, whilst PpαF has the capacity for post-translational translocation in yeast, it is an obligate substrate of the co-translational pathway in a mammalian in vitro system [23]. The mature domain of PpαF has three endogenous sites for N-linked glycosylation [24], whilst we employed a version of PpCecA that is tagged at its C-terminus with an N-terminal fragment of opsin bearing either one (PpCecAOPG1) or two N-linked glycosylation sites (PpCecAOPG2) (Fig. 1A, see I and IV; [16]). The resulting N-glycosylated species were identified by comparing the products synthesized in the presence and absence of ER derived microsomes (see Fig. S1); and by confirming the sensitivity of presumptive N-glycosylated species to treatment with Endoglycosidase H (see Fig. S2). On this basis, we subsequently used the molecular weight shift resulting from the N-glycosylation of imported proteins to be used as a reporter for translocation into the ER lumen.

We synthesized PpCecAOPG2, PpαF and PCecApαF in vitro, either in the presence of ER derived membranes, i.e. “co-translational” addition, or prior to their post-translational addition, and then analyzed the resulting products for evidence of ER translocation. In the isolated membrane fraction, singly and doubly glycosylated forms of PpCecAOPG2 can be observed, consistent with import into ER microsomes under both “co-” and “post-translational” conditions (Fig. 1B, lanes 1 and 2). PpαF is N-glycosylated upon import into the ER and all three potential glycoforms are visible when the protein is synthesized in the presence of membranes, albeit the triply N-glycosylated form predominates (Fig. 1B, lane 5; see also Fig. S1, panel C). However, when membranes are added after translation is complete, the glycosylated species is barely detectable, indicating that the protein is not efficiently translocated under these experimental conditions (Fig. 1B, lane 6). When the signal sequence of PpαF is exchanged for the PpCecA signal sequence, PCecApαF retains the ability to be translocated during ongoing synthesis but is unable to be efficiently translocated across the membrane post-translationally (Fig. 1B, lanes 3 and 4). Interestingly, the replacement of the PpαF signal with that of PpCecA also appears to result in the under glycosylation of the mature region of the protein, with the majority of the polypeptides appearing to have only two N-linked glycans (Fig. 1B, cf. lanes 3 and 5). This observation may reflect inefficient signal cleavage from the chimeric protein, a conclusion that is supported by analysis with SignalP 4.0 [25] and consistent with the observation that perturbation of signal peptidase function also results in the hypoglycosylation of PpαF at a site proximal to the signal sequence [26] (cf. Fig. 1A, II). Furthermore, although a faster migrating mature form of PpαF, consistent with the efficient processing of its signal sequence, can be clearly distinguished after deglycosylation (Fig. S2, panel C) this was not the case for PCecApαF (Fig. S2, panel D). Hence, either signal sequence cleavage from PCecApαF is inefficient, and/or the cleaved pro-form and uncleaved Prepro-form of the protein have an almost identical mobility upon SDS-PAGE. On the basis of these data we conclude that the PpCecA signal sequence alone is not sufficient to permit efficient post-translational translocation of the PCecApαF chimera.

To determine whether regions other than the signal sequence of PpCecA might contribute to its ability to be post-translationally translocated, we analyzed a second chimera where the PpCecA signal sequence was replaced by that of PpαF (PαFpCecAOPG1, see Fig. 1A, IV). The two parent proteins, PpCecAOPG1 and PpαF, together with this chimera, were again translocated in vitro under either “co-translational” or “post-translational” conditions. Strikingly, PαFpCecAOPG1 is efficiently N-glycosylated under post-translational conditions, indicating that the PpαF signal sequence can support the post-translational translocation of the mature procecropin A domain (Fig. 1C, cf. lanes 3 and 4). Taken together, these data indicate that the PpαF signal sequence has the capacity to support post-translational translocation across mammalian ER microsomes, but that the properties of the mature domain of the polypeptide that it delivers to the ER can affect the translocation process.

### The Sec62 Dependency of pCecA Translocation is Signal Sequence Independent

**Figure 2:**
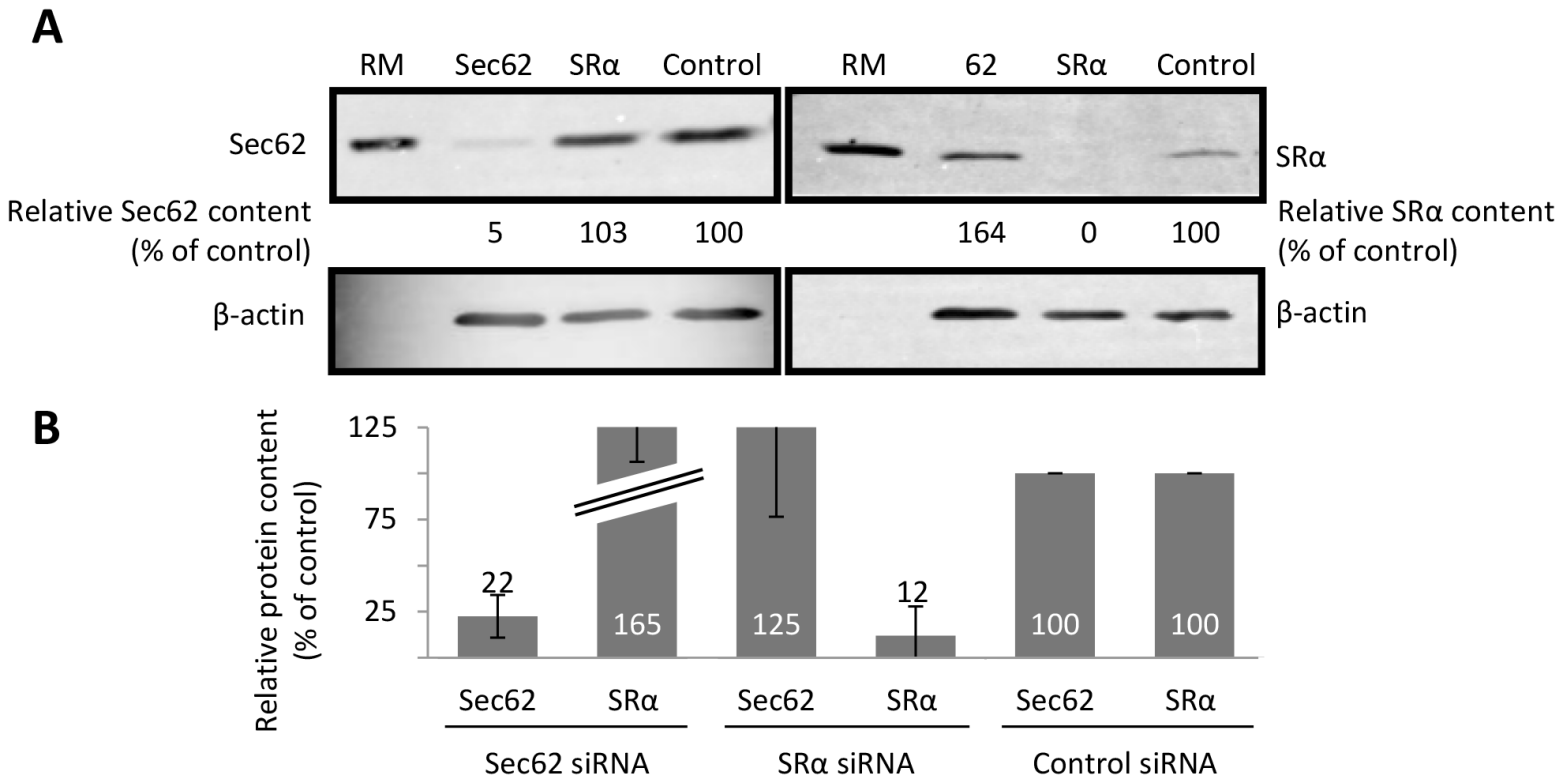
siRNA mediated depletion of ER membrane components. HeLa cells were treated with a control siRNA, or siRNAs against Sec62 (A), SRα (A) and Sec63 (B), subsequently harvested, and the relative levels of specific proteins determined by SDS-PAGE and immunoblotting. Levels of β-actin were used as a loading control and, following quantification, the relative amount of reach component was expressed as a percentage of the siRNA control sample. The values in the accompanying graphs show means (±s.e) for n ≥3, and in some diagrams the upper section of the error bars is omitted to preserve a uniform format.

In order to provide insight into the molecular mechanisms that differentiate precursors translocated co- or post-translationally, we studied the role of specific ER membrane components. Both co- and post-translational translocation occur via the Sec61 translocon [18], and we therefore focused on the role of upstream components that most likely act as “receptors”. The SRP receptor (SR) is a well-established component of the co-translational pathway for translocation [2], [4], whilst the mammalian Sec62/63 complex has recently been implicated in the post-translational translocation of short secretory proteins [18], [19]. We selectively depleted these components using siRNA treatment of mammalian cells and employed western blotting to confirm substantial reductions in the level of specific components (Figs. 2A and 2B, see also [18]). Following depletion, the cells were then treated with digitonin to generate semi-permeabilised cells suitable for in vitro translocation assays [18], [27], [28]. For these studies, we employed conditions that allowed “co-translational” translocation; that is, semi-intact cells were present during protein synthesis, and we relied on the depletion of specific cellular components to perturb, and thereby distinguish, translocation specific pathways.

**Figure 3:**
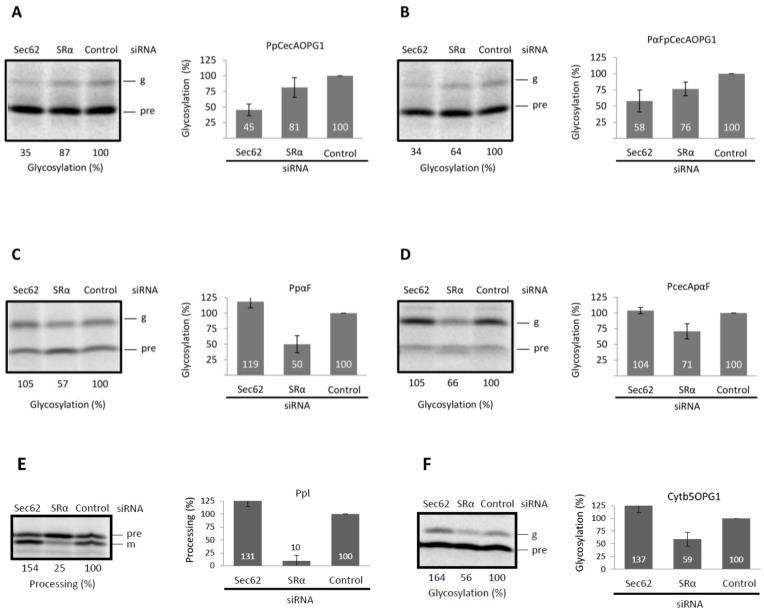
Protein translocation into the ER of cells depleted of Sec62 and SRα. HeLa cells were depleted of Sec62 or SRα by siRNA treatment and subsequently permeabilised with digitonin to enable ER translocation of in vitro synthesized proteins. The following protein precursors were translated in vitro in the presence of these semi-intact cells: PpCecAOPG1 (**A**), PαFpCecAOPG1 (**B**), PpαF (**C**), PCecApαF (**D**), Ppl (**E**) and Cytb5OPG1 (**F**). Membranes were isolated by centrifugation and associated proteins were analyzed by SDS-PAGE and phosphorimaging. Translocated polypeptides were identified on the basis of the N-glycosylation of the mature region, except for Ppl where signal sequence cleavage was used as an alternative reporter. In each case, the translocated products were each quantified relative to the control sample, which was set to 100%. All results are shown as means (±s.e) for n ≥3.

Although the translocation of short secretory proteins into the ER of semi-permeabilized cells was noticeably less efficient than that observed with canine pancreatic microsomes (cf. Figs. 1B and 1C), the siRNA-mediated depletion of Sec62 resulted in a reproducible reduction in the translocation of PpCecAOPG1 and PαFpCecAOPG1 (see Figs. 3A and 3B). Based on the level of substrate N-glycosylation, this reflected a ∼50% decrease in protein translocation as compared to control cells (Fig. 3A and 3B). In contrast to these two short precursor proteins, the translocation of PpαF, PCecApαF and the co-translational paradigm, preprolactin (Ppl), were either unaffected (Figs. 3C and 3D), or potentially even enhanced (Figs. 3E), by Sec62 depletion. This reflects the strongly “co-translational” behavior of these substrates in the presence of ER microsomes (Fig. 1, see also [23]) and suggests they are incapable of using post-translational alternatives for ER delivery. In further support of this hypothesis, PpαF, PCecApαF and Ppl all show clear reductions in translocation efficiency, ranging from ∼30–90%, upon depletion of the SRα subunit of the SRP receptor (Figs. 3C–E), indicating a substantial reliance on the SRP dependent pathway for ER delivery. Interestingly, PpCecAOPG1 and PαFpCecAOPG1 also show some reduction in translocation upon SRα depletion, albeit less severe. Taken together, these results suggest that a substantial proportion of these two short secretory proteins exploit a Sec62 dependent pathway for ER delivery, although a smaller fraction of each may also be targeted to the ER via an SRP dependent, and hence most likely “co-translational” route. In contrast, the post-translational integration of the tail-anchored membrane protein cytochrome b5 (Cytb5OPG1) is unaffected by Sec62 depletion (Fig. 3F), consistent with the prevailing view that it is inserted independently of the Sec61 complex [17], [18]. However, SRα depletion does perturb the membrane insertion of Cytb5OPG1 (Fig. 3F), and hence perhaps mammalian SRP can also act post-translationally [29].

### The Dependency of Protein Translocation on Sec63 Mirrors that for Sec62

**Figure 4:**
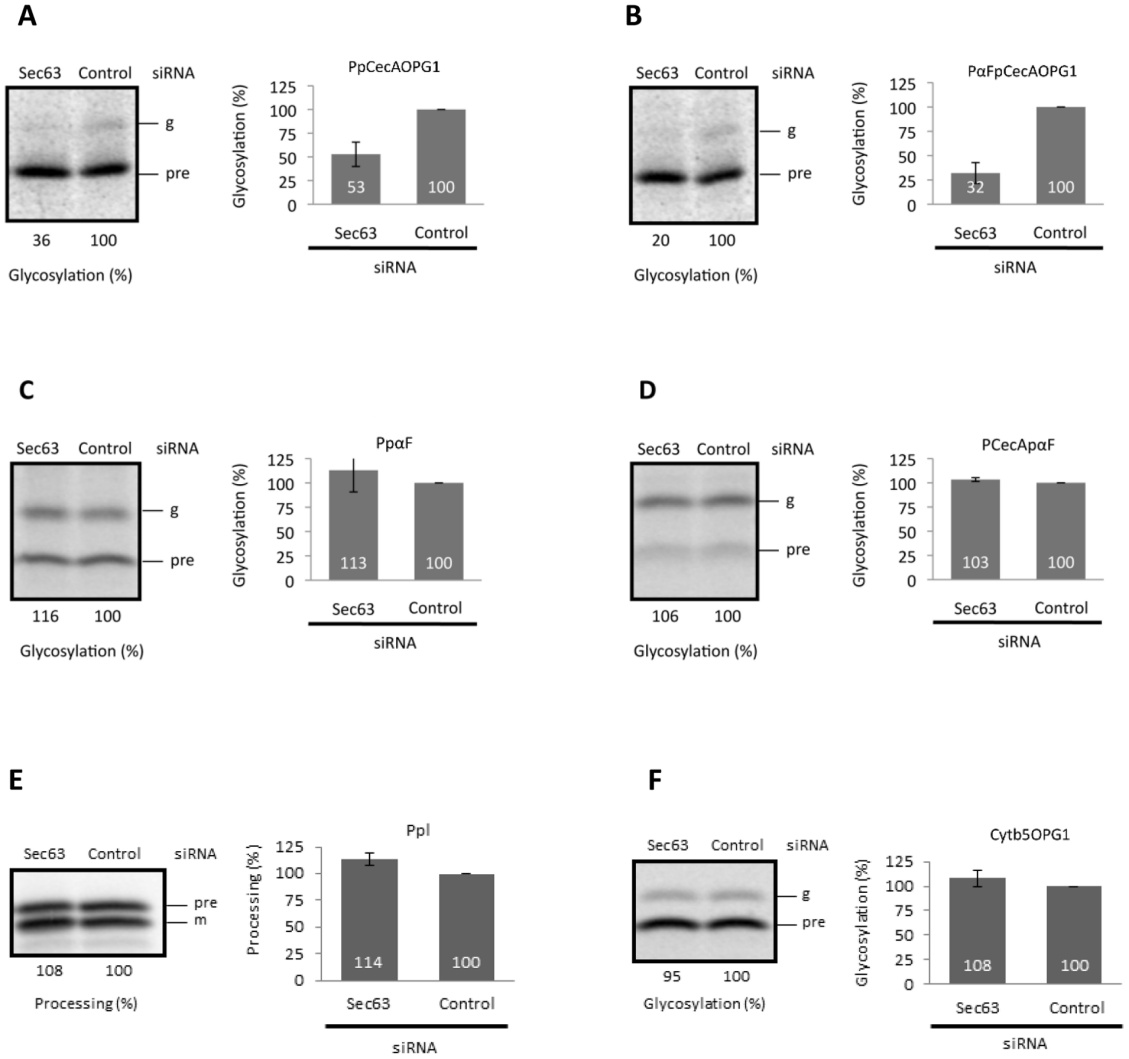
Protein translocation into the ER of cells depleted of Sec63. The precursors shown in Figures 3A to 3F were synthesized in the presence of semi-intact cells depleted of Sec63 and analyzed as previously described. The labeling of the precursors (panels **A** to **F**) is as shown for Figure 3, and all results are given as means (±s.e) for n = 3.

A rather similar pattern of precursor protein behavior is observed with mammalian cells that have been depleted of Sec63 prior to their use in ER translocation assays. As before, these experiments were performed under conditions where the proteins were synthesized in the presence of semi-permeabilised cells to enable “co-translational” translocation. Under these circumstances, the translocation of the two short secretory proteins, PpCecAOPG1 and PαFpCecAOPG1, is reduced by ∼50 to 70% (Figs. 4A and 4B). In contrast, the translocation of the three longer precursor proteins (>160 residues) PpαF, PCecApαF and Ppl is unaffected by the siRNA mediated reduction in Sec63 (Figs. 4C to 4E). Likewise, the post-translational integration of Cytb5OPG1 is also comparable in control and Sec63 depleted cells (Fig. 4F), consistent with previous studies [18]. Based on the secretory proteins studied here, Sec62/63 dependent precursors appear to utilize these components irrespective of the origin of their signal sequence, be it “post” (PpCecA) or “co” (PpαF). This in turn suggests that some other property dictates the selection of a Sec62/Sec63 dependent translocation pathway by precursors, for example the chain length of the protein [19].

### Signal Sequence Specific Engagement of the Sec61 Translocon is Ribosome Independent

**Figure 5:**
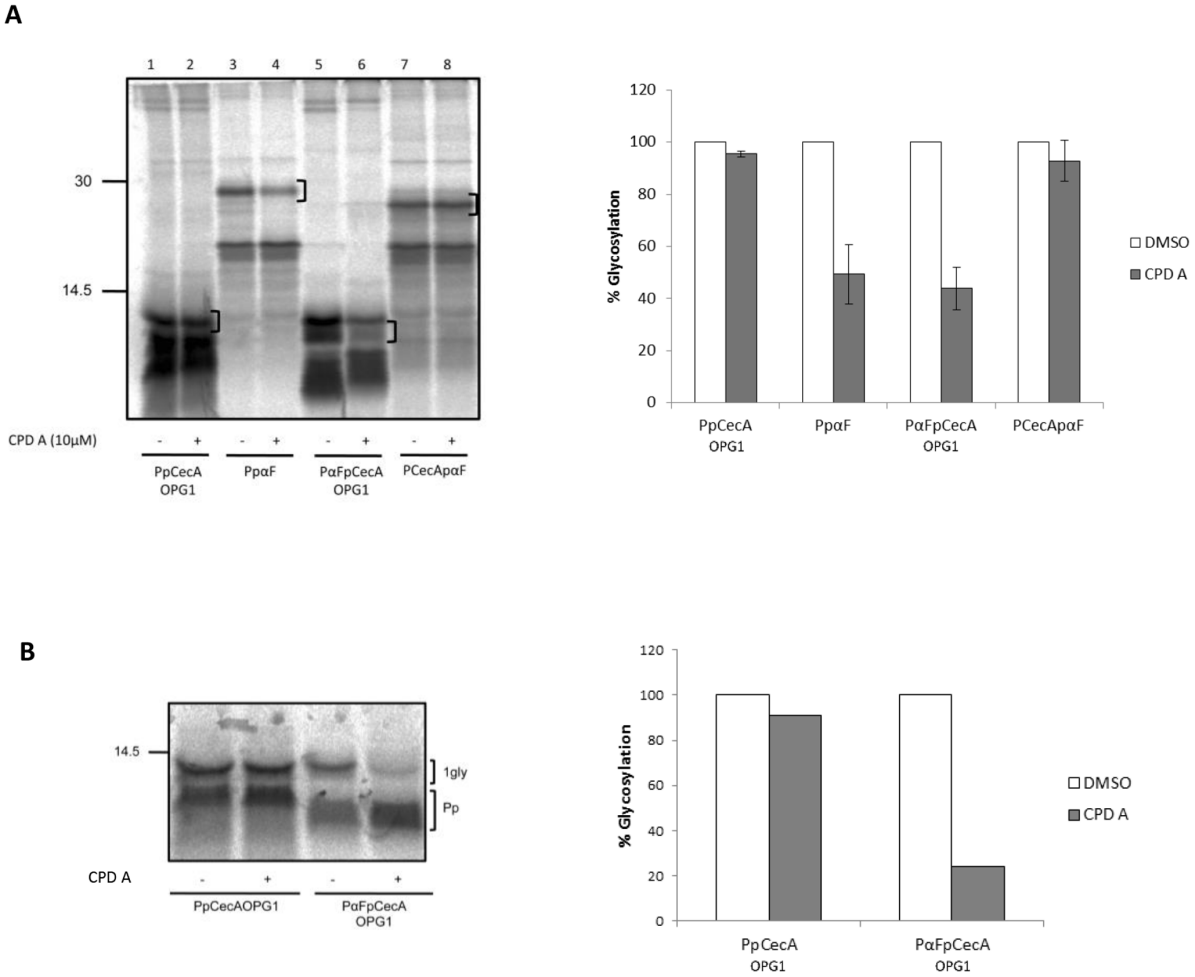
The signal sequence modulates post-translational interactions with Sec61. **A.** PpCecAOPG1, PpαF and the two chimeras, PαFpCecAOPG1 and PCecApαF were translated in the presence of ER microsomes and either DMSO (−) or 10 µM CPD A (+). Membranes were isolated by centrifugation and associated proteins were analyzed by SDS-PAGE and phosphorimaging. A bracket indicates the predominant glycoform, and this species was quantified and expressed as a percentage of the paired DMSO treated samples which was set to 100%. The values shown are means (±s.e) for n = 3. In this experiment, PαFpCecAOPG1 has generated two products upon translocation into the ER, both of which are reduced upon treatment with CPD A concomitant with an apparent increase in the prepro-form of the protein that retains its signal sequence (cf. lanes 5 and 6). PαFpCecAOPG1 has only a single N-linked glycan and the two forms observed are most likely a consequence of glucose trimming [40]. **B.** The post-translational translocation of PpCecAOPG1 and PαFpCecAOPG1 into ER derived microsomes pretreated with DMSO (−) or CPD A dissolved in DMSO (+) was performed by adding membranes after protein synthesis had been terminated using puromycin (see also materials and methods) and then processing the samples as described for A. Glycosylated products shown in panel B were quantified by phosphorimaging and expressed as a percentage of their paired DMSO sample.

Since both SR and Sec62/63 are proposed to facilitate the transfer of precursors to the Sec61 complex, we next studied the influence of the signal sequence at this stage of the translocation process by screening our chimeric proteins using a selective inhibitor of Sec61 mediated co-translational translocation. This inhibitor, referred to here as compound A (CPD A), has previously been shown to selectively inhibit Sec61 dependent co-translational translocation in a signal sequence dependent fashion [30], [31], [32]. For this experiment, all proteins were initially synthesized in the presence of membranes (i.e. “co-translational translocation”) so that the effect of the compound on all four precursors could be directly compared. PpCecA, PpαF and our two chimeric precursors were synthesized in the presence of membranes and 10 µM CPD A or solvent alone. Both PpαF and PαFpCecAOPG1 translocation was clearly inhibited in the presence of the compound (Fig. 5A, lanes 4 and 6), whilst PpCecA and PCecApαF were completely unaffected (Fig. 5A, lanes 2 and 8). This suggests that CPD A selectively inhibits the translocation of proteins bearing the signal sequence from PpαF.

The effect of CPD A on the post-translational translocation of proteins at the Sec61 complex has not been investigated, and we took advantage of our two PpCecA derived precursors to address this issue. Strikingly, we continue to observe a signal sequence specific inhibition of Sec61 mediated protein translocation even under conditions of strictly post-translational translocation (Fig. 5B, cf lanes 1 to 4). We conclude that CPD A acts to specifically perturb the engagement of the PpαF signal sequence with the ER translocon, and consequently inhibit the translocation of proteins that bear it, irrespective of whether the precursor is delivered via a co- or post-translational mechanism. This in turn implies that neither the association of a nascent polypeptide chain with the ribosome, nor the binding of the ribosome to the Sec61 translocon, play any role in the signal sequence specific inhibitory action(s) of CPD A (see also Discussion).

### Both Signal Sequence and Delivery Pathway can Influence BiP Dependency of Translocation

**Figure 6:**
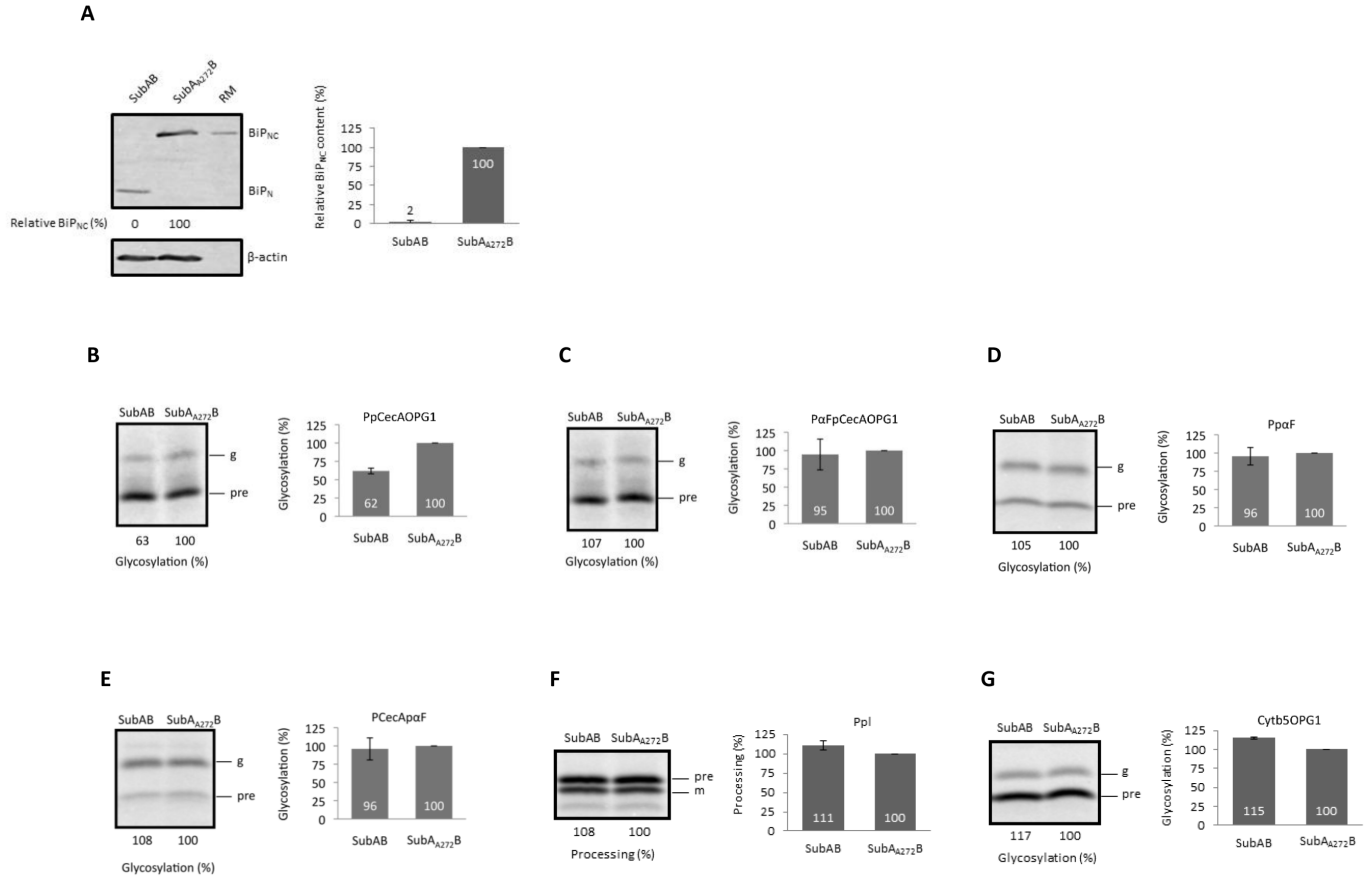
The requirement for BiP is signal sequence dependent. **A**. HeLa cells were pre-incubated with recombinant SubAB or its inactive mutant, SubA_A272_B, and the resulting BiP cleavage confirmed via a molecular weight shift from the full-length form (BiP_NC_) to a lower molecular weight species (BiP_N_). The intact BiP content of cells treated with the active form of SubAB is expressed relative to those treated in parallel with the inactive mutant, and the accompanying graph shows the means (±s.e) for n = 3. **B** to **G**. HeLa cells were treated with the cytotoxin AB_5_ subtilase (SubAB) or an inactive mutant (SubA_A272_B), precursor proteins (as Figures 3 and 4) synthesized in the presence of the resulting semi-intact cells, and samples analyzed by quantification as before (see Figures 3 and 4). All results are given as means (±s.e) where n = 3 (panels **F** and **G**), n = 4 (panels **B** and **C**) or n = 5 (panels **D** and **E**).

Molecular chaperones of the ER lumen, most notably BiP, can promote the translocation of polypeptides through the Sec61 complex [33], [34]. To address the potential role of BiP in facilitating the translocation of our model precursors, we employed the cytotoxin AB_5_ subtilase (SubAB) in order to achieve an acute perturbation of BiP function. Hence, mammalian cells were pretreated with SubAB, or an inactive mutant form of the protein, SubA_A272_B, for two hours, enabling the toxin to reach the ER lumen and cleave and inactivate BiP [22]. Immunoblot analysis confirmed both the effectiveness of the SubAB treatment and the inactivity of the SubA_A272_B mutant (Fig. 6A). We therefore proceeded to study the effect of BiP inactivation upon the translocation of our model secretory protein precursors by semi-permeabilizing the toxin treated cells and using them for in vitro translocation assays. Strikingly, we find that PpCecAOPG1 translocation is reproducibly reduced by ∼40% following treatment with the active form of SubAB (Fig. 6B), indicating that its efficient import is dependent upon BiP activity, consistent with a previous study that implicated BiP in PpCecA translocation [34].

In contrast, the translocation of PαFpCecAOPG1 was unaffected by the same treatment (Fig. 6C), despite the ability of both precursors to exploit a post-translational translocation pathway into the ER lumen (Fig. 1) and their strong dependency on Sec62 and Sec63 (cf. Figs. 2 and 3). This effect cannot be attributed solely to the signal sequence present on the two short secretory proteins, since when the same signal sequences are analyzed in the context of the longer pαF mature region, the translocation of both PαFpCecA and PCecApαF are unaffected by SubAB treatment (Figs. 6D and 6E). Likewise the translocation of Ppl, and the membrane integration of the tail-anchored protein Cytb5OPG1 are comparable upon pre-treatment of cells with the active or control toxin (Figs. 6F and 6G). On the basis of these data, we conclude that the potential contribution of BiP to the efficient translocation of a secretory protein precursor is a function of both the signal sequence responsible for its ER delivery, and its capacity to exploit a co- or post-translational mechanism for membrane translocation.

## Discussion

**Figure 7:**
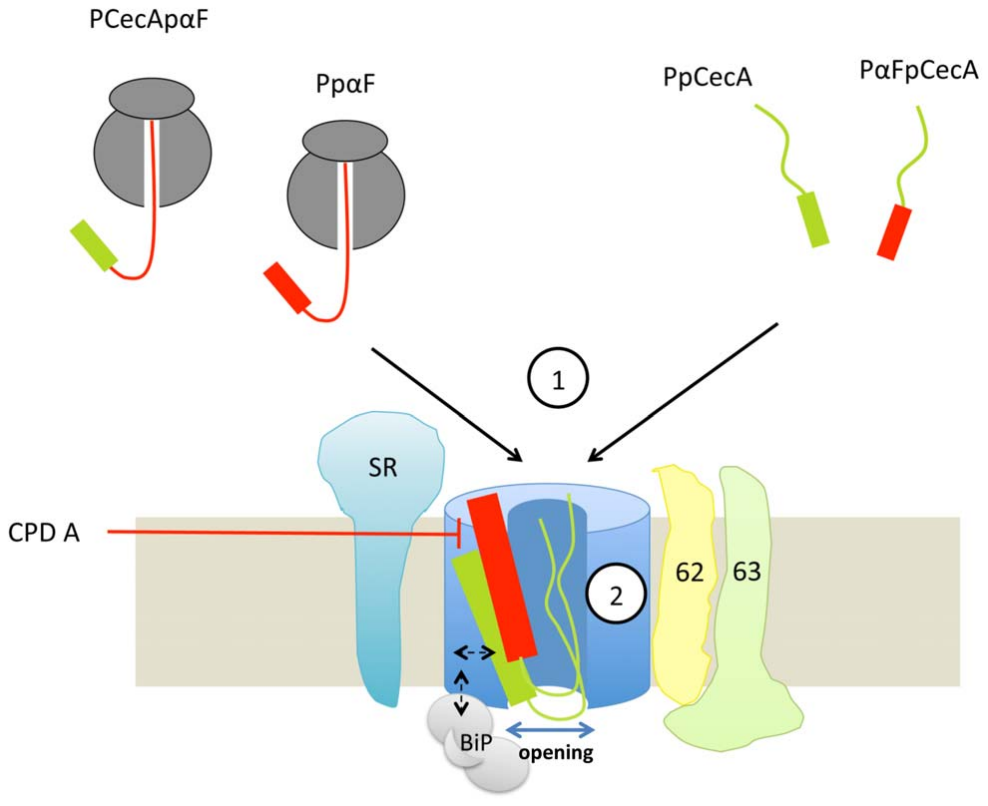
A model for distinct routes across the ER translocon. Large proteins (on the left) are co-translationally translocated across the ER in a process dependent upon the SRP receptor complex (SR). Short proteins (on the right) utilize a post-translational route(s) that require the Sec62/63 complex. Both routes are independent of the properties of the signal sequence (1). However, the signal sequence appears to affect how the precursor engages with the translocon both co- and post-translationally (2), as reflected in differing sensitivity to CPD A. The signal sequence may also determine whether BiP is required for the efficient translocation of short secretory proteins. Dashed arrows suggest how a specific mode of engagement of the signal sequence with the translocon might enable BiP to support translocation.

Our results show that the PpCecA signal sequence alone is not sufficient to enable the post-translational translocation of any passenger protein, consistent with previous studies of chimeras derived from PpCecA and preprolactin [15], [35]. Furthermore, our studies of the PαFpCecAOPG1 chimera indicate that the capacity of a precursor for post-translational translocation is most likely dictated by the properties of its mature domain since, in this case, the exchange of its signal sequence did not substantially diminish its capacity for post-translational translocation in vitro (see Fig. 7). Our analysis suggests that chain length may be one important parameter in relation to the ability of a precursor protein to exploit a post-translational translocation pathway across the ER membrane [14], [19]. In short our study using a well-established cell free system suggests that the mature region of PpCecA remains competent for efficient post-translational translocation when placed behind heterologous signal sequences that are capable of mediating the ribosome independent ER delivery of precursor proteins (Fig. 7).

Consistent with the hypothesis that diverse ER signal sequences can enable the post-translational translocation of an appropriate passenger protein, in this case a short secretory protein precursor (∼80 residues), we find that the efficient translocation of both PpCecA and PαFpCecA are strongly dependent upon both Sec62 and Sec63 (Fig. 7). These are two Sec61 translocon-associated components that were first defined in yeast as playing an important role in post-translational translocation [4], and more recently shown to play a comparable role in mammals [18], [19]. Whilst we favor a model where the effect of Sec62 and Sec63 perturbations upon PpCecOPG1 and PαFpCecAOPG1 translocation reflect their use of a post-translational pathway, it should be noted that these mammalian components also display ribosome-binding activity [36]. Since our experiments using semi-permeabilized cells were carried out under conditions that allow both co- and post-translational translocation, we cannot exclude the possibility that Sec62 and Sec63 may also facilitate the co-translational translocation of these two substrates via a pathway that acts independently of SRP and the SRP receptor.

In contrast to the results we obtained using PpCecAOPG1 and PαFpCecAOPG1, when both signal sequences are delivered to the ER in the context of the longer pαF mature domain (>160 residues) the previously observed requirement for Sec62 and Sec63 is completely lost (Fig. 7). Rather, these longer precursors now show a strong reliance on the SRP receptor, consistent with their SRP dependent co-translational delivery to the ER as ribosome bound nascent chains [37]. A relationship between precursor chain length and the cellular components that facilitate ER translocation has recently been observed with several other proteins, where increasing polypeptide chain length correlated with a decreasing reliance on Sec62 and an increasing dependency on soluble SRP and its integral membrane protein receptor [19]. Our current study now expands this hypothesis to include Sec63, which we speculate most likely acts in concert with Sec62, as previously observed for the equivalent components in yeast [4].

Intriguingly, although the strongest perturbation of their ER translocation occurs upon the depletion of Sec62 and Sec63, our data also suggest that a proportion of PpCecA and PFpCecAOPG1 can use an SRP dependent pathway. Hence, depletion of the α-subunit of the SRP receptor results in a ∼20–25% reduction in translocation. Whilst the precise molecular basis for this effect remains to be determined, it may be that even short secretory protein precursors enjoy a brief “window of opportunity” to exploit the SRP dependent co-translational pathway. These findings support the proposal that a significant proportion of the PpCecA can employ both co- or post-translational routes in vivo [15], and provide direct evidence for an SRP dependent contribution to the biosynthesis of short secretory proteins (cf. [19]). It is also possible that some short secretory proteins may exploit an SRP dependent delivery pathway that is post-translational, as previously suggested for selected tail-anchored membrane proteins [29]. Irrespective of the precise molecular details of their ER delivery, it appears that multiple soluble components can facilitate the post-translational delivery of precursors to the ER [5], [8], [15], [16]. However, upon the arrival of short secretory proteins at the ER, our data suggest that Sec62 and Sec63 play an important role in their subsequent translocation [18], [19].

Experiments with CPD A demonstrate that the PpαF signal sequence is sensitive to this compound but the PpCecA signal is not. Previous work has clearly demonstrated that CPD A interferes with the interaction of certain signal sequences with the core ER translocon component, Sec61α [30], [31], [32]. Although we can only speculate as to the precise molecular mechanisms underlying the differences we observe, our data are consistent with a model where the engagement of the two signal sequences with the translocon is distinct (Fig. 7), perhaps reflecting differences in their intrinsic hydrophobicity [31], [32], [38]. These observations build on previous studies of the yeast ER translocon that also suggested a signal sequence recognition or proof-reading step takes place at the Sec61 complex [39].

Strikingly, we also find evidence that the presence of a specific signal sequence can influence the requirement for BiP during the translocation of the precursor. This effect was only apparent in the context of a Sec62/63 dependent precursor and we speculate that this enhanced role for BiP is most likely a reflection of the post-translational route for ER translocation that is followed by PpCecAOPG1 but not by the longer PCecApαF precursor. Exactly how a signal sequence acts in this context remains to be determined, but this region might mediate some priming event at an early stage of the translocation process that is linked to the role(s) of BiP [34]. Hence, variations in signal hydrophobicity or differences in translocon engagement, as revealed by sensitivity to CPD A treatment, may affect pore opening or other events influencing translocation efficiency, thereby resulting in a dependency on BiP (Fig. 7). In summary, our data suggest that whilst the presence of a signal sequence is essential for efficient protein targeting to the ER, the specific delivery pathway/receptor utilized is dictated by the length of the mature region, resulting in either an SR dependent co-translational pathway or a Sec62/63 dependent pathway that we propose is most-likely to be post-translational [18]. Regardless of the delivery route employed, the properties of the signal sequence can directly influence both precursor engagement of the Sec61 complex and at least one additional, BiP-dependent, translocation promoting step.

## Supporting Information

Figure S1 **Translocation dependent products of PpCecA, PpαF and their respective chimeras.** PpCecAOPG1, PpCecAOPG2, PpαF and the two chimeras, PαFpCecAOPG1 and PCecApαF (see Fig. 1) were synthesized in the presence (RM) or absence (Buffer) of ER microsomes as indicated. At the end of the translation reaction total samples (all precursors synthesized without ER microsomes and PpαF and PCecApαF synthesized with ER microsomes), or isolated membrane fractions (PpCecAOPG1, PαFpCecA and PpCecAOPG2 synthesized with ER microsomes), were subjected to sequestration analysis by treatment with Proteinase K in the presence and absence of Triton-X100. The resulting samples were subjected to SDS-PAGE and visualized by phosphorimaging. The untranslocated (Pp), singly, doubly and triply N-glycosylated (1 g, 2 g, 3 g) and signal cleaved (p) forms of the various precursors are indicated.
(TIFF)

Figure S2 **Confirmation of N-glycosylated forms of PpCecA, PpαF and respective chimeras using Endoglycosidase H treatment.** PpCecAOPG1, PpCecAOPG2, PpαF and the two chimeras, PαFpCecAOPG1 and PCecApαF were synthesized in the presence of ER microsomes. Total products (PpαF and PCecApαF), or isolated membrane fractions (PpCecAOPG1, PαFpCecA and PpCecAOPG2), were subjected to Endoglycosidase H (EndoH) treatment, followed by SDS-PAGE and phosphorimaging. The untranslocated (Pp), singly, doubly and triply N-glycosylated (1 g, 2 g, g3) and signal cleaved (p) forms of the precursors are indicated. The asterisk indicates a PCecApαF derived product of unknown origin that is most likely a partially de-glycosylated species.
(TIFF)
